# A tailored intervention for the detection of patients with coronary heart disease and mental or cognitive comorbidities in the German primary care setting: qualitative evaluation of implementation success

**DOI:** 10.1186/s12913-024-11841-z

**Published:** 2024-11-22

**Authors:** Christin Herrmann, Belinda Werner, Florian Wurster, Ute Karbach, Charlotte Leikert, Laura Nordmeyer, Adriana Meixner, Lena Sannemann, Christian Albus, Frank Jessen, Ludwig Kuntz, Frank Schulz-Nieswandt, Holger Pfaff, on behalf of the Cologne Research and Development Network (CoRe-Net) Study Group, Christian Albus, Frank Jessen, Ludwig Kuntz, Frank Schulz-Nieswandt, Holger Pfaff, Ingo Meyer, Nadine Scholten, Stephanie Stock, Julia Strupp, Raymond Voltz

**Affiliations:** 1 Chair of Quality Development and Evaluation in Rehabilitation, Faculty of Human Sciences & Faculty of Medicine and University Hospital Cologne, Institute of Medical Sociology, Health Services Research and Rehabilitation Science, University of Cologne, Cologne, Germany; 2 Faculty of Management, Economics and Social Sciences, Institute of Sociology and Social Psychology (ISS), University of Cologne, Cologne, Germany; 3 Department of Psychosomatics and Psychotherapy, Faculty of Medicine and Cologne University Hospital, University of Cologne, Cologne, Germany; 4 Department of Business Administration and Health Care Management, Faculty of Management, Economics and Social Sciences, University of Cologne, Cologne, Germany; 5 Department of Psychiatry and Psychotherapy, Faculty of Medicine and Cologne University Hospital, University of Cologne, Cologne, Germany; 6 German Center for Neurodegenerative Diseases (DZNE), Bonn, Germany; 7 Excellence Cluster on Cellular Stress Responses in Aging-Associated Diseases (CECAD), University of Cologne, Cologne, Germany; 8 Centre for Health Services Research Cologne (ZVFK), Faculty of Medicine and Cologne University Hospital, University of Cologne, Cologne, Germany

**Keywords:** Implementation, Coronary heart disease, Primary care, Implementation science, Knowledge translation, Qualitative, Mental and cognitive disorder

## Abstract

**Background:**

Guidelines recommend the identification of potential mental and/or cognitive disorders (MCD) in patients with coronary heart disease (CHD). However, compliance with these guidelines appears to be lacking in primary care. A minimal invasive intervention was tailored with experts for the primary care setting to increase the identification of this patient group and ensure proper treatment. The intervention includes: A trigger question, screening tests and question prompt sheet for patients. Following the implementation of this intervention in primary care physician (PCP) offices, the aim of this study is to evaluate the implementation outcomes.

**Methods:**

Semi-structured interviews were conducted with ten PCPs who tested the intervention for six months. The study was guided by Proctor’s Framework on Implementation Outcomes to understand the appropriateness, feasibility, acceptability, fidelity and sustainability of the intervention as proxies for implementation success.

**Results:**

Relevance of the topic and the need for the intervention is recognised by all of the PCPs. All PCPs were willing to try the intervention and considered it generally appropriate and feasible. Additionally, supporting implementation resources were considered helpful in familiarising with the intervention. Screening of patients with a first diagnosis of CHD, those who have had experienced a recent coronary event and those who have been hospitalised for CHD is considered practical and appropriate. Known barriers such as lack of knowledge, perceived relevance and awareness were successfully addressed. It was not possible to overcome barriers such as time pressure, forgetfulness, and patient reaction. Additionally, the paper format of the information materials was perceived as impractical, and integration into the physician information system was identified as a possible way to increase acceptance. Nevertheless, PCPs stated they will continue to be aware of the link between CHD and MCD and want to maintain their individualised approach.

**Conclusions:**

The study provides important insights into the use of a minimal invasive intervention in primary care. Despite tailoring the intervention to the primary care setting, implementation success was suboptimal due to individual barriers in PCP offices. This highlights the need for tailored approaches at the level of individual PCP offices to better address context-specific barriers.

**Supplementary Information:**

The online version contains supplementary material available at 10.1186/s12913-024-11841-z.

## Background

Coronary heart disease (CHD) is a significant global health problem affecting people predominately aged 50 years and older [1]. In addition, CHD is the leading cause of death worldwide and also in Germany [2–4]. The comorbidity between mental and cognitive disorders (MCD) is well established. Mental disorders include conditions such as depression and anxiety, and cognitive disorders include cognitive impairment and dementia. The prevalence of depression in patients with CHD is 15–30%, which is two to three times higher than in the general population [5]. There is strong evidence that both depression and anxiety adversely affect CHD’s disease prognosis. They increase the risk of mortality [5–7] and reduce an individual’s quality of life [8]. They also decrease the adherence to important medical treatments and necessary lifestyle changes [9]. In addition, studies have documented associations between CHD and cognitive disorders such a cognitive impairment or dementia. For example, cognitive deficits have been found to be associated with decreased adherence to treatment [10], which could have a negative impact on CHD’s disease prognosis. Conversely, CHD has been shown to increase the risk of cognitive impairment or dementia [11, 12]. Therefore, current guidelines recommend regular screening and appropriate treatment of comorbid MCD in patients with CHD [6, 9, 10, 13]. Primary care physicians (PCP) are the main actors which are responsible for the application of this guidelines and the appropriate detection of MCD comorbidities [14].

Despite recommendations, the adherence to screening and treatment in routine care appears to be inadequate [14–19]. A study by Peltzer et al. showed that PCPs could correctly identify and diagnose only about half of CHD patients with MCD [16]. There was also a notable deficit in terms of comprehensive treatment for psychological distress while primarily treating the CHD [16].

A well-known problem is the discrepancy between scientific knowledge and its adequate use in practice [20–22]. This gap leads to suboptimal patient care, unnecessary treatment, and costs to individuals and the health care system [20]. It also remains a critical problem in mental health services [23]. Implementation science aims to close this gap by integrating scientific evidence into health care, but translating research into practice remains a complex challenge [24, 25]. Effective treatment and effective implementation are distinct aspects and implementation is a prerequisite for achieving desired improvements in clinical and service outcomes [26].

A minimal invasive (MINI) intervention was developed to contribute to the implementation of the guideline recommendations into daily practice. The primary aim of the intervention was to increase PCP awareness of the comorbidity of CHD and MCD. The secondary aim was to improve the detection and management of MCD in patients with CHD in primary care. The MINI intervention includes a trigger question (TQ) and screening tests for PCPs and a question prompt sheet for CHD patients.

In a multi-stage process, the MINI intervention is tailored to the specific needs of a primary care setting: (1) a literature review served as the basis, (2) expert interviews (ten PCPs, seven CHD patients and three patient representatives) were conducted to identify relevant determinants using a framework [27] (3) the research team prioritised the determinants that could be addressed in the pilot study, and (4) strategies were derived to address these determinants [28]. The determinants to be addressed and the corresponding strategies are summarised in Table 1. The identified determinants have also been identified in systematic reviews as key factors for behavioural change among PCPs and for interventions in primary care setting [29–31].

**Table 1:** Determinants and strategies

Domain	Determinate	Strategy	Consideration
Primary care physician	Reminder of use of the intervention	Design reminder tool	Coat pocket card, Tear-off sheet card
	Lack of knowledge about comorbidity of CHD and MCD	Provide evidence of comorbidity in CHD and MCD patients, red flags	Training course Booklet
	Relevance and awareness of comorbidity of CHD and MCD	Communicate needs and value to patients and PCPs	Training course Booklet
Primary care office	Rigid structures	Support in adapting the routine	Training course MINI Intervention Coat pocket card, Tear-off sheet card
	Limited time	Keep the intervention as short as possible; Training course in their practice	Training course MINI Intervention
Patient	Need for support and information	Provide information	Question prompt sheet
	Inhibitions to address CHD impairments	Encourage patient to talk openly about impairments; Sensitise PCP	Training course MINI Intervention Question prompt sheet
	Stigmatisation	Educate patients; Sensitise PCP	Training course Question prompt sheet
Intervention	Complexity of the intervention	Structured, simple process	MINI Intervention

Tailoring interventions to address identified barriers is more likely to improve professional practice rather than simply disseminating guidelines [32]. The tailored intervention was tested over a six-month period in selected PCP offices in Cologne, Germany.

The current study is part of a larger project designed as a pilot study, following the principles for the development and evaluation of complex interventions [33]. The larger project tailored the MINI intervention and evaluated its feasibility at several levels: The effect of the MINI intervention on changes in PCP behaviour, organizational characteristics on patient-level outcomes, cumulative socioeconomic return, and evaluation of implementation outcomes [28].

The latter is the subject of this study, which focuses specifically on the experiences of PCPs in using the tailored MINI intervention in their daily routines. A variety of methods are available for the evaluation of implementation [26, 34–36]. In this study, the evaluation was conducted using Proctor’s Framework of Implementation Outcomes [26], which provides a framework for measuring the success of implementation processes. Proctor defines implementation outcomes as “the effects of deliberate and purposive actions to implement new treatments, practices, and services” [26]. The results of this study will provide a deeper understanding of the implementation success of the MINI intervention, its challenges in use and its subjectively perceived benefits.

## Methods

### Minimal invasive intervention

The MINI intervention has been designed as a two-sided intervention (PCP and patient) and includes several elements: A TQ and screening tests for PCP and a question prompt sheet for patients. In this context, “minimal invasive” refers to an intervention that hardly interferes with routine care practice.

**Fig. 1:**
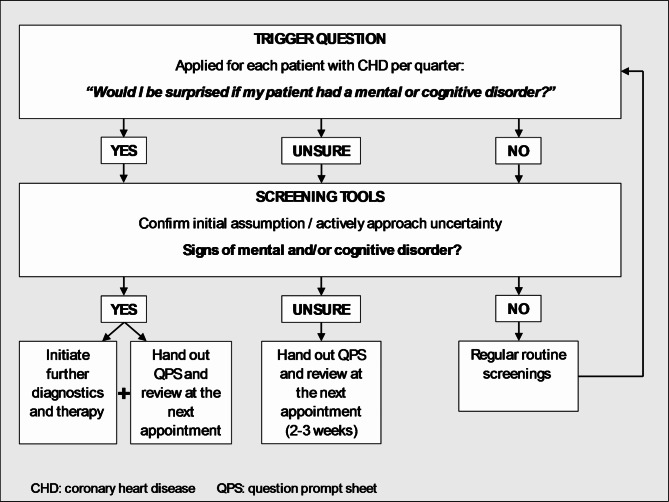
MINI intervention: Flow chart for PCPs as a guide for using the trigger question, screening test and the question prompt sheet output

The **Trigger Question (**TQ) “Would I be surprised if my patient had a mental or cognitive disorder?” is designed to encourage reflection on the decision-making process and to challenge routine assumptions and behaviours. PCPs should ask themselves the TQ at least quarterly for each patient with CHD and confirm their assumption of the presence of an MCD or actively manage uncertainty by using screening methods. Depending on the screening results, they should follow up with further diagnostic, therapeutic interventions and provide information to the patient. Figure 1 illustrates this process.

PCPs have been provided with **Screening Tools** for time-efficient identification. Cognitive impairment screening includes a question about subjectively perceived cognitive decline and related concerns and the Six-Item-Screener [37]. Depression or anxiety screening begins with an open-ended question about the patient’s current feelings, followed by the Patient Health Questionnaire for Depression and Anxiety (PHQ-4) [38], and concludes with another open-ended question about any other mental health problems.

For patients, a **Question Prompt Sheet** (QPS) has been developed to provide appropriate support for communication between patients and PCPs. Its use can empower patients to take an active role in communicating with their PCP and ask questions, thereby increasing their knowledge and satisfaction, improving the doctor-patient relationship [39–41] without increasing consultation time [40, 42, 43]. The QPS provides information on the comorbidity of CHD and MCD, examples of relevant questions, space for personal notes and information on further resources or contacts.

### Supporting implementation resources

To support implementation of the MINI intervention, a training course and information materials were provided to PCPs. A 90-minute in person **training course** was conducted in the PCPs’ offices to increase their knowledge and awareness of the common comorbidity of CHD and MCD and to familiarise them with the intervention. The training course was led by senior investigators (CA, FJ) who are specialists in psychosomatic medicine and psychiatry and have many years of experience in CHD and PCP research, and used a specially developed presentation as a guideline to ensure consistent training and reliability. **Information material** were designed specifically for PCPs participating in the study. A booklet provided detailed information on the latest guideline recommendations for the detection and management of MCD in patients with CHD and further guidance on the intervention. In addition, coat pocket cards (Supplementary file 1) contained procedural and screening tools, while tear-off sheets (Supplementary file 2) allowed for documentation of test results in the patient’s record. Further details of the MINI intervention and supporting implementation resources are reported in the study protocol [28].

### Study design

This study is a part of OrgValue II, a study on characteristics of value-based care from the perspective of care institutions and MenDis-CHD II, a study on quality of care in diagnosis and therapy of MCD in CHD. Both are a part of the interdisciplinary Cologne Research and Development Network (CoRe-Net), a competence network of practice and research for the model region of Cologne, Germany [44]. CoRe-Net, as well as OrgValue II and MenDis-CHD II, was funded by the German Federal Ministry of Education and Research (BMBF). The whole project was based on the value-based health care approach [45–47]. The study has been approved by the Ethics Commission of the Faculty of Medicine of Cologne University (ID 21-1530). It has been registered at the German Clinical Trials Register under the ID DRKS00022154 (Registration Date: 02 November 2021). The manuscript reporting was guided by Standards for Reporting Qualitative Research (SRQR) [48].

The MINI intervention was implemented in seven PCP offices in Cologne, both individual and group offices. Recruitment took place via several channels, including presentation of the project at PCP events or symposia, recruitment via CoRe-Net, and invitation e-mails or letters to local PCP networks and PCP offices associated with the University Hospital Cologne. The participating PCPs had no advanced training in basic psychosomatic care and no additional specialist or additional qualification in psychotherapy. All selection criteria and recruitment of participating PCP offices are detailed in the study protocol [28]. After a six-month intervention period, during which PCPs had the opportunity to try out the intervention and integrate it into their daily practice, a qualitative interview was conducted with each participating PCP.

This study evaluated the implementation outcomes of the MINI intervention using the Proctor´s Framework for Implementation Outcomes [26]. The operationalised definitions of the implementation outcomes reported in this study are shown in Table 2 (appropriateness, feasibility, acceptability, fidelity and sustainability).

**Table 2:** Operationalised definitions of proctor´s implementation outcomes

Implementation Outcome	Operationalised Definitions
Appropriateness	*Appropriateness* is defined as the perceived fit, relevance and compatibility of the MINI intervention with the PCP office setting and stakeholder involved (PCP, medical assistants, patients), as well as the perceived potential of the MINI intervention to address an existing problem.
Feasibility	*Feasibility* is defined as the extent to which the MINI intervention can be successfully used and implemented as intended in PCP office, taking into account compatibility and available resources.
Acceptability	*Acceptability* is defined as the perception of the stakeholder involved in practice (PCP, medical assistants, patients) that the MINI intervention is agreeable and satisfactory, e.g. in terms of content, visual appearance and complexity
Fidelity	*Fidelity* is defined as the extent to which the MINI intervention was used in a daily practice as intended by the developers.
Sustainability	*Sustainability* is defined as the intention to use the MINI intervention in the future.

### Data collection

An interview guide (supplementary file 3) eliciting implementation outcomes according to Proctor et al. [26] was developed and presented to a qualitative research circle for review by other researchers. Each topic was operationalised by core questions facilitating story-telling and narrative-generating sub-questions. In addition, the PCPs were encouraged to speak freely about their experiences with the intervention, with the guide being flexibly adapted to the interview process. Depending on the preference of the general practitioner, the interviews took place either in the PCP office or virtually. Data were collected between December 2022 and April 2023. All interviews were conducted by the first and second authors, who are trained interviewers. Both had no previous contact with the PCPs. A professional transcriber transcribed the audio recordings of the interviews verbatim.

### Data analysis

The semi-structured interviews were analysed using qualitative content analysis. This allows the perspective of the interviewees to be accurately reflected and the content to be systematically described [49]. The interview transcripts were managed in MAXQDA©, a computer-assisted qualitative data analysis software by VERBI in Berlin, Germany. The coding tree (supplementary file 5) was created based on the questions in the interview guide and expanded using a combination of deductive and inductive approaches [49]. The analysis was carried out in three steps:

1. Coding process: All transcripts were coded independently by two researchers (CH, BW). Inductive categories and inconsistencies were then discussed until consensus was reached (consensus coding). This procedure ensures that all relevant information has been identified in the data material and the validity of the data interpretation.
2. Assignment of categories to Proctor´s implementation outcomes: All deductive and inductive categories were assigned to the five implementation outcomes. The content of each category was thoroughly reviewed to determine if it matched the implementation outcome.
3. Data summary: A summary was created for each case in a within-case matrix that included the five implementation outcomes and their corresponding categories. These summaries were then merged into a cross-case matrix. The cross-case matrixes is to observe outcomes across multiple cases, understand how these outcomes are influenced by local conditions, and generate more detailed descriptions and powerful explanations [49]. (Please refer to supplementary file 4 for an exemplary extract from the cross-case matrix.) All analysis steps required several meetings within the research team.

## Results

### Sample

The MINI intervention to detect MCD in patients with CHD was tested by 12 PCPs from 7 PCP offices over a six-month period. Interviews were conducted with *N* = 10 PCPs. At least one PCP from each of the seven participating PCP offices was included in the study. The remaining two PCPs retired from practice for personal reasons and did no longer take part in the study. The appropriateness, feasibility, acceptability, fidelity and sustainability of this intervention were evaluated. In addition, barriers and suggestions for improvement expressed by PCPs were reported. Quotes from PCPs have been included to illustrate the results.

### Appropriateness

Regarding the appropriateness of the intervention, the relevance of the issue was recognised by all PCPs and the intervention was generally considered useful. The intervention has the potential to reduce stigmatisation of cognitive and mental impairment among patients if used on a long-term basis and has helped to raise awareness among PCPs about MCD in patients with CHD.

*So this topic was not so- maybe not so important for me earlier. I thought CHD is a difficult diagnosis. I didn’t interpret it as an anxiety disorder or depression or or or. And*,* so I am actually more sensitised.* (PCP 7)

According to the PCP’s subjective assessment, there were more discussions about MCD with patients during the intervention period.

*What I did more often as a result is simply to start a conversation. So*,* somehow asking out of the conversation*,* how are you dealing with it now*,* and what has changed for you now*,* or do you see life differently*,* or whatever.* (PCP 9)

Although only a few patients with MCD requiring treatment were identified, there were also cases of mild disorder that did not require immediate medication or referral to a specialist, but were monitored subsequently by the PCP. If abnormalities were found during screening, appropriate care was initiated (e.g. referral to neurology or psychosomatic specialist; recommendation for psychotherapy; prescription of medication). However, PCPs noted that referring patients to specialists was challenging due to a shortage of therapy places, resulting in longer waiting times.

*Of course*,* I first tried to find a place for psychotherapy. That has NOT yet worked out*,* and I have already started antidepressants with one patient.* (PCP 7)

The intervention was considered appropriate for the primary care setting, but PCPs reported some limitations. They considered full screening for not appropriate for all patients with CHD and recommended its targeted use for those with a first diagnosis of CHD, recent hospitalisation for CHD, or a recent coronary event, and additionally suggested further screening for patients reporting cognitive or mental impairment.

### Feasibility

In terms of feasibility, the intervention was considered theoretically feasible, but practical barriers were identified. PCPs noted that time constraints make it impossible to perform the MINI intervention and full screening in all patients with CHD.

*In the routine of a general practice with a high patient volume*,* it is sometimes not practical. It is then difficult to implement. But otherwise*,* if you had the time for it*,* it would be easy. But under time pressure*,* it just fizzles out.* (PCP 5)

The usual time frame for a patient contact was not sufficient for an adequate discussion of MCD. PCPs emphasise the importance of a trusting relationship for a sensitive discussion of MCD, and that both approaching the patient and discussing MCD take time.

*Many people need longer contact before they talk about any issues. And they don’t trust us because they see a different doctor here every week. And that’s also a point. With my regular patients*,* patients I know well*,* it works quite well*,* but with others*,* unfortunately not.* (PCP 7)

There were also challenges on the part of PCPs, who reported that it became increasingly difficult to remember the MINI intervention, especially for appointments for acute health problems e.g. influenza symptoms. Another barrier was the lack of feedback on referrals made or from continuing care psychotherapists or psychiatrists, which also led to a decrease in initial motivation during the intervention phase.

*So you try to initiate it. How efficient that is*,* what impact it has on treatment*,* that is not always clear to me. You don’t see the people that often neither. Some you see once. And then they don’t come back for months. And you don’t follow up*,* I don’t call again the one I sent to the psychologist.* (PCP 2)

Patient-related challenges were also described by the PCPs. Addressing potential cognitive and mental impairments was a challenge for some PCPs, in part because they feared that this would lead to problems. In addition, patients sometimes reacted with surprise and irritation, or even directly defensiveness, when asked about their mental and cognitive well-being.

*I would say 90% of the patients we addressed were always surprised. It was ‘why should I’- or often the question was ‘Why are you asking me this now?.* (PCP 4)

The PCPs identified potential improvements to increase the feasibility of the MINI intervention, including shortening the screening to 2–3 specific questions, recommending a self-administered questionnaire for patients, and the involvement of medical assistants. In addition, the intervention could be better integrated into regular appointments, such as a routine quarterly appointment in a disease management program (DMP) for CHD.

*So maybe it will be easier if you actually include it as another question in the DMP questions. It would be no extra effort.* (PCP 4)

PCPs who implemented the intervention as planned were surprised that, despite the intensive enquiries, no more patients reported impairment. PCPs suggest that routinely addressing mental and cognitive well-being may normalise the topic. Patients may come to see the recurring questions as a regular part of the consultation, leading to more open discussions about possible impairment.

### Acceptability

In terms of acceptability, the interviews indicated that the structured process and information materials were overall helpful and supportive in familiarising PCPs with the intervention. Supporting patients in this situation with a QPS was also seen as an added value.

*Not leaving the patient alone but giving them something as a follow-up. So that they feel there is something structured. It’s a very good idea*. (PCP 10)

The training course was rated as helpful, low-threshold and interesting, with content that raised awareness of the comorbidity between CHD and MCD.

*It [training course] was a good meeting*,* which was very content-focused and really sensitized you to it*,* opened up a few facets that I hadn’t realized before.* (PCP 1)

However, acceptability in daily practice was hampered by the fact that the pre-written screening questions were not easily integrated into the conversation or were not in the PCPs` language, and the use of slips of paper such as the coat pocket card and tear-off sheets during a consultation was seen as unusual and confusing to patients. PCPs suggested that integrating the information materials into the physician information system, e.g. pop-up reminders, would make them easier to use and remember in daily practice, potentially increasing acceptance.

*If I pull out a block and just start ticking boxes in a conversation with the patient sitting across from me at close quarters*,* they get the impression that this is now a standardised process. The personal attention and focus is distracted by these cards. […] So I think if it goes through the physician information system*,* the practice computer*,* and you see the diagnosis*,* maybe a pop-up at the bottom saying ‘psychological problem*,* sleep disorder’*,* that would be a very good hint.* (PCP 1)

There is criticism that there is no structured reimbursement for routine screening of all patients with CHD outside the study design. From the perspective of PCPs, integrating certain elements of the intervention into the CHD DMP could be a solution to compensate for the additional time required outside the study design. Overall acceptability was average. It could be increased by adjusting the aspects of appropriateness and feasibility that were perceived as negative.

### Fidelity

The study did not formally measure adherence and accuracy of use. Rather, the approach described by PCPs was compared with the original MINI intervention as intended by the study team. In terms of fidelity, the interviews showed that all PCPs were willing to try the intervention. The aim was to screen all patients with CHD, but PCPs adapted their approach individually and according to the situation. Information on the parts of the intervention and how they were used and adapted by the PCPs is available in Table 3.

**Table 3:** Usage and adaption of the intervention by PCP

**Trigger Question Usage by PCPs**	• **Used as Intended**: 1 PCP for all CHD patients and additional groups • **Adapted Use**: o 1 PCP used TQ only for patients with a recent myocardial infarction or non-ST elevated myocardial infarction o 1 PCP used TQ only for patients attending the CHD DMP o 4 PCPs used TQ more as a reminder and posed their own question to the patient, e.g. “Well, how are you? Do you have any complaints?” o 1 PCP rephrased TQ as “What impression does the patient make?”, “Could he/she have anything?” • **Not Used:** 2 PCPs
**Screening Usage by PCPs**	• **Used as Intended**: 1 PCP for all CHD patients • **Adapted Use**: 7 PCPs adapted screening individually and according to the situation, specific details include: o Screening conducted occasionally, if symptoms were reported o Screening was often shortened by only addressing individual questions or topics o Modifications in the wording of questions to better integrate into consultations. o 2 PCPs used other screening instruments: Dementia detection test, Mini-Mental-Status-Test • **Not Used**: 2 PCPs
**Booklet**	• **Used as Intended**: 3 PCPs used the booklet at least once after the training for reference • **Not Used**: 7 PCPs
**Coat Pocket Cards**	• **Not Used**: No PCP used the coat pocket cards.
**Tear-off Sheets**	• **Partially Used**: 4 PCPs used the tear-off sheets o included 1 PCP gave the sheet to the patients to complete themselves o included 1 PCP placed the sheets in the paper file to be able to compare the results in case of a repeat • **Not used**: 6 PCPs
**Question Prompt Sheet for Patients: Issuance by PCPs**	• **Issuance as Intended**: 1 PCP to all patients with CHD • **Partially Issuance**: 7 PCPs regularly or occasionally to patients • **Not issued**: 2 PCPs • **Displayed in Waiting Room**: 7 PCP offices also displayed the QPS in the waiting room

### Sustainability

Sustainability is defined here as the intention to continue using the MINI intervention after the end of the study. PCPs reported that they would continue to be aware of MCD and CHD even after the study ended. The majority of PCPs indicated that they would maintain their individual approach, use the information materials as needed and continue to display the QPS for patients in the PCP offices.

*I will incorporate it better into my everyday life*,* so the questions and document them in the medical record. And then do the tests specifically when I feel it’s necessary.* (PCP 4)

## Discussion

The aim of this study was to evaluate the implementation success of a tailored intervention aimed primarily to increase PCP awareness of the comorbidity of CHD and MCD, and secondarily to improve the detection and management of MCD in patients with CHD in primary care. Interviews with PCPs were used to gather subjective experiences of this MINI intervention, to gain a deeper understanding of the success of implementation, the challenges of using it in primary care, and the subjectively perceived benefits.

### Subjectively perceived benefits

The results of the study show that the PCPs interviewed generally recognised the relevance of the topic and generally considered the MINI intervention to be useful. The relevance of comorbidity of CHD and MCD was not known or present to most of the PCPs in this study. This is supported by the findings of Feinstein et al. [50] who found that half of the PCPs surveyed were not aware that depression itself can be an important risk factor for CHD. Karbach et al. also showed that only 40% of PCPs were sufficiently aware of cardiovascular disease guidelines that recommend asking patients about mental comorbidity [51]. PCPs emphasise that awareness of MCD in CHD patients has increased and that they pay more attention to symptoms in daily practice. It should be emphasised that the training course specifically provided PCPs with knowledge about of the comorbidity of CHD and MCD and made them aware of the extent and the problems associated with it. It also effectively addressed the barrier of awareness and knowledge.

Based on current research, it can be assumed that depression and anxiety [15, 16, 52] and cognitive impairment and dementia [53–55] are underdiagnosed in primary care. It was therefore expected that increased awareness of these problems by PCPs in the study would lead to an increased detection rate in patients. According to PCPs own assessment, this has been limited, with only a very small number of patients identified as needing treatment for MCD. This may be because the intervention was not used as intended. In the majority of practices, not all CHD patients were screened, with some PCPs relying on their first impression of the patient’s health and softening the wording of the questions. So, it is possible that patients did not see a reason to talk about symptoms.

The MINI intervention is designed as a two-sided intervention, making the patient a “co-producer”, meaning that active participation is crucial to the success of the intervention [56]. Detection of MCD impairments relies heavily on patient cooperation. Without open and honest responses, it is difficult for PCPs to assess the patient’s true health status. Stigmatisation of mental and cognitive impairments is known to be a widespread problem in society, resulting in patients being ashamed to talk openly about their impairments [57–60]. Other studies also report that patients’ rejectionist attitudes make it more difficult to recognise cognitive impairments [61]. From the PCPs’ perspective, the intervention has the potential to reduce this barrier of patient stigmatisation. This suggests that regular discussion of impairments will normalise the topic and reduce irritation or surprise. Based on the routine examinations within the CHD DMP [62], the CHD patient had 1–2 appointments with the PCP during the six-month intervention period. This may not be sufficient to establish a sense of normality.

### Variation in the use of the MINI intervention

The MINI intervention was seen as a simple, structured process, and the information materials were helpful in familiarising PCPs with the intervention. This successfully overcame the barrier of the complexity of the intervention. But it was not used by all PCPs as intended. The main challenge is the limited consultation time, which does not allow for an adequate discussion of MCD. According to Irving et al., the average time spent consulting a patient in German primary care is 7.6 min [63]. Acceptance of screening all patients with CHD, as envisaged in the intervention, is low. This is partly because it is not considered appropriate and partly because of the limited consultation time, especially for acute symptoms. Bradford et al. confirm that consultations in primary care are typically short and patients often present themselves with several different symptoms [61]. The barrier of limited time could not be overcome despite the tailored intervention process. Despite the structured nature of the MINI intervention and short screening, it is not possible to screen all patients with CHD in routine primary care. Other studies also show that time is a significant barrier to screening and prevention interventions in primary care [31, 64].

It is therefore considered useful to narrow the target group to patients with a first diagnosis of CHD, those who have been hospitalised for CHD and those who have had a recent coronary event. These patients are acutely confronted with the consequences of the event or CHD, which is an appropriate time to sensitise them to possible MCD. It is also likely that there is an increased need for information at this time of the disease.

In general, there should be a separate, designated setting for such discussions about CHD and related symptoms and problems. The CHD DMP was mentioned several times as an existing structure that could be used for this purpose. It is conceivable that a shortened screening program (with 2–3 questions) could be carried out within the DMP to ensure better thematic and time integration.

It is reasonable to assume that all participating PCPs were initially motivated to use the MINI intervention, given that PCPs participated in the study on a voluntary basic. However, there was a tendency for initial motivation to decrease during the intervention phase. Reasons given were lack of feedback from psychotherapists or psychiatrists providing further treatment and lack of response from patients. Motivation of PCPs is a key factor in the successful implementation of prevention and health promotion activities [65]. It is central to changing one´s practice [66], and perceived positive changes in practice increases the likelihood of establishing new routines [67]. Positive changes were perceived by PCPs only to a very limited extent, which may explain the diminishing motivation and the limited use of the intervention. Building on initial motivation alone was not enough. Targeted measures should have been taken to maintain initial motivation throughout the intervention period.

Another barrier was remembering to carry out the MINI intervention. This was mainly due to difficulties in adapting to their existing routines, the stress of daily practice, and particularly when consulting for acute symptoms. Information materials, such as the coat pocket card and tear-off sheets, which were designed to remind PCPs of the intervention and help them adjust their routines, proved ineffective. Although these materials were initially perceived as helpful, dissatisfaction with the paper format eventually prevailed as it was considered impractical in a primary care setting. A systematic review by Cheung et al. still supports this approach, highlighting that reminders are an effective way to improve health professional behaviour and the quality of care [68]. To overcome the limitations of paper materials, digitalisation of content or integration into the physician information system, along with pop-up reminders, would likely be more effective. In support of this, a study of computer reminders for practices adhering to guideline recommendations for haemoglobin and glucose management showed a significant effect on adherence to the recommendations. This suggests that computer reminders are valuable tools to support implementation [69].

Although each barrier is described separately, they occur in daily practice as overarching and interdependent challenges. The barriers of reimbursement, limited capacity and lack of treatment places are outside the scope of this study and were not directly addressed, although they were mentioned in the interviews. These factors are also widely discussed in the literature as important determinants. A systematic review of barriers to effective management of mental health problems in children and adolescents concluded that inadequate reimbursement and a lack of providers and resources were the main barriers for primary care practitioners in recognizing and diagnosing mental health problems in young people [70].

As PCPs consider the intervention to be useful in terms of the outcome appropriateness, the variability in implementation could be explained by the logics of standardisation and individualisation [71]. Within the logic of standardisation, specific barriers hinder the planned implementation, even though individuals are willing to adhere. Individualisation, which runs counter to this logic, is characterised by a strong sense of autonomy on the part of the individual, which means that although an intervention proposed from the outside would be feasible, it is often not implemented successfully because of the individual’s desire for self-determination. One solution could be to merge the concepts of individualisation and standardisation [56]. Individualised standardisation of care is defined as “the imposition of standards, regulations or norms which are tailored to the genes, body condition, culture, social environment, values, needs and preferences of the individual patient” [72]. The approach uses context management that makes individuals with a strong sense of autonomy more receptive to control signals. Within this context, treatment must be individualised despite standardisation, which allows for justified deviations from guidelines [56].

Barriers were identified in advance and taken into account in the tailoring of the MINI intervention. However, while some barriers were successfully overcome, others remained unresolved. The identification of barriers focused on the setting of primary care. These findings, combined with the concept of individualisation, indicate that future projects should tailor interventions not only to the setting, but also to the specific context of the PCP´s office in order to meet their needs for autonomy.

## Strengths and limitation

There are a variety of methods for evaluating implementation of interventions [34, 36]. The results of the present study suggest that the chosen qualitative approach to collect PCPs experiences of implementing the MINI intervention is a suitable approach to evaluate subjectively perceived benefits and feasibility [35, 73]. In addition, the guidance by Proctor´s Implementation Outcomes provides a deeper understanding of the specific dynamics and challenges associated with implementing the MINI intervention in daily practice while ensuring connectability with further research, also relying on Proctor´s Implementation Outcomes.

The small sample size represents a limitation of this study. However, the consistency of the themes expressed by the participants suggests that data saturation was achieved. Especially when analysing experiences and perspectives within a relatively homogeneous group, a small sample can be sufficient to achieve comprehensive data saturation [74, 75]. However, in order to complete the sample, it would have been desirable to interview the two PCPs who did not participate until the end of the study.

In terms of the larger project (OrgValue II and MenDis-CHD II), the qualitative data alone are not sufficient to make a final assessment of feasibility. For a comprehensive evaluation, the effects of the MINI intervention and the patient perspective must also be considered. Although a final judgement cannot yet be made, it is important to report these findings as they provide valuable insights.

## Conclusions

Overall, the results indicate that PCPs are more aware of the comorbidity of CHD and MCD. According to their own assessment, PCPs were only able to identify a very small number of patients requiring treatment for MCD, but the initiation of specific therapies in these cases indicates a potential improvement in patient care. There is a potential for an increase in detection rates if the intervention is used for more than six months, but this cannot be conclusively answered with the data available at this point. It also has the potential to reduce patient stigma if used regularly. The results suggest that comprehensive screening of all patients with CHD, as originally planned, is not appropriate. Instead, it is recommended that the intervention should be used specifically for first diagnosis of CHD, for those who have been hospitalised for CHD and for those who have had a recent coronary event, and to be integrated into routine consultations, e.g. in the CHD DMP to check for MCD.

In conclusion, the study highlights the importance of tailoring interventions and implementation strategies to the specific context of the PCP’s practice. Even within the primary care setting, there may be relevant differences. Therefore, to maximise implementation success, the planning and implementation of the intervention should be tailored to the specific practice context, taking into account both, the challenges of implementing the intervention in a primary care setting and the specific needs of patients with CHD.

## Electronic Supplementary Material

Below is the link to the electronic supplementary material.

Supplementary Material 1.

Supplementary Material 2.

Supplementary Material 3.

Supplementary Material 4.

Supplementary Material 5.

## Data Availability

The transcripts used and analysed in the current study are not publicly available in order to protect the anonymity of participants, and the content may compromise confidentiality. An anonymised version of the data can be provided by the corresponding author upon reasonable request.

## Abbreviations

MCD: Mental and cognitive disorders
CHD: Coronary heart disease
PCP: Primary care physicians
TQ: Trigger question
QPS: Question Prompt Sheet
DMP: Disease-Management-Program
