# Supplementary material for: A tailored intervention for the detection of patients with coronary heart disease and mental or cognitive comorbidities in the German primary care setting: qualitative evaluation of implementation success
Source: BMC Health Serv Res. 2024 Nov 22;24:1454. doi: 10.1186/s12913-024-11841-z (PMC11585210; doi:10.1186/s12913-024-11841-z)
Supplement: Supplementary file 1 — Supplementary Material 1. [file 12913_2024_11841_MOESM1_ESM.pdf]

Screening: Hinweise zur Identifikation einer psychischen und / oder kognitiven Störung

Anwendung der
TRIGGER QUESTION
bei allen KHK-Patient*innen pro Quartal

„Wäre ich überrascht, wenn mein*e Patient*in an einer kognitiven und / oder psychischen Störung leiden würde?"

↓ **Ja** | ↓ **Bin mir unsicher** | ↓ **Nein**

Annahme bestätigen bzw. Unsicherheit aktiv klären
➔ Screening-Fragen (Kittelkarte / Abreißblock) stellen

Hinweise für psychische und / oder kognitive Störung?

↓ **Ja**

- Weitergehende Diagnostik und Therapie
- \+ Ausgabe QPS; Klärung beim Folgetermin

↓ **Bin mir unsicher**

- Ausgabe QPS; Klärung beim Folgetermin (ca. 2-3 Wochen)

↓ **Nein**

- Regelmäßiges Screening im Verlauf (→ zurück zur Trigger Question)

CoRe-Net

Screening für psychische Störungen (Depression, Angst, Sonstige)

1. Offene Einstiegsfrage: „Wie fühlten Sie sich in den letzten Wochen in seelischer Hinsicht?“

2. Gesundheitsfragebogen für Patient*innen (PHQ-4)[1]:
„Wie oft fühlten Sie sich im Verlauf der letzten 2 Wochen durch die folgenden Beschwerden beeinträchtigt?“

Skala	Item	Überhaupt nicht	An einzelnen Tagen	An mehr als 50% der Tage	Beinahe jedenTag
**Depression** (PHQ-2) = Summe der Items 1 und 2, Scores von ≥ 3 sind klinisch relevant.	1: Wenig Interesse oder Freude an Ihren Tätigkeiten	0	1	2	3
	2: Niedergeschlagenheit, Schwermut oder Hoffnungslosigkeit	0	1	2	3
**Angststörung** (GAD-2) = Summe aus den Items 3 und 4, Scores von ≥ 3 sind klinisch relevant.	3: Nervosität, Ängstlichkeit oder Anspannung	0	1	2	3
	4: Nicht in der Lage sein, Sorgen zu stoppen oder zu kontrollieren	0	1	2	3

3. Offene Anschlussfrage: „Haben Sie noch weitere seelische Beschwerden außer den bisher genannten?“

Auffälligkeiten? ➔ **Leitliniengerechte Versorgung und Ausgabe des Question Prompt Sheet**

CoRe-Net

Screening für Demenz und Mild Cognitive Impairment

1. Subjektive Gedächtnisprobleme

„Haben Sie das Gefühl, dass Ihr Gedächtnis in letzter Zeit nachgelassen hat?“

➔ Bei positiver Antwort gefolgt von:

↓

„Machen Sie sich deswegen Sorgen?“

2. Six-Item Screener[2]

„Ich nenne Ihnen 3 Dinge. Bitte warten Sie, bis ich alle 3 Wörter gesagt habe, und wiederholen Sie sie dann. Merken Sie sich die Wörter, denn ich frage Sie demnächst noch einmal danach. Bitte wiederholen Sie:“

(1. Wort) – (2. Wort) – (3. Wort) z. B. „Auto - Blume - Ball“

(Maximal 3 Lernversuche ➔ alle 3 Wörter richtig wiederholt?) **Ja** ○ **Nein** ○

➔

CoRe-Net

„Jetzt stelle ich Ihnen 3 Fragen:“

**„Welches Jahr haben wir?“**	○ **korrekt = 1**	○ **nicht korrekt = 0**
**„Welchen Monat haben wir?“**	○ **korrekt = 1**	○ **nicht korrekt = 0**
**„Welchen Wochentag haben wir?“**	○ **korrekt = 1**	○ **nicht korrekt = 0**

„Wie hießen die 3 Dinge, die Sie sich merken sollten?“

**(1. Wort)**	○ **korrekt = 1**	○ **nicht korrekt = 0**
**(2. Wort)**	○ **korrekt = 1**	○ **nicht korrekt = 0**
**(3. Wort)**	○ **korrekt = 1**	○ **nicht korrekt = 0**

Anzahl der erreichten Punkte: ___ / 6

(Bei Erreichen von 5 oder 6 Punkten sind alltagsrelevante kognitive Störungen mit hoher Wahrscheinlichkeit ausgeschlossen).

Auffälligkeiten? ➔ **Leitliniengerechte Versorgung und Ausgabe des Question Prompt Sheet**

CoRe-Net

Relevante Belastungsfaktoren erkennen

für die Entstehung psychischer / kognitiver Störungen bei einer KHK oder somatischer Komplikationen im Verlauf der Erkrankung

Risikofaktoren	Mögliche Fragen	Empfehlungen
**Niedriger sozioökonomischer Status** bezogen auf:[3]<br>Bildung, Einkommen, Beruf, Wohnort.[4]	„Was ist Ihr höchster Bildungsabschluss?“[3]<br>„Was ist Ihr Beruf?“[3]	Niedrigschwelliger Zugang zu kardiovaskulären Präventions-Angeboten
**Beruflicher Stress**[3]	„Haben Sie das Gefühl, dass Sie Kontrolle über die Anforderungen in Ihrem Beruf haben?“[5]<br>„Erhalten Sie deutlich zu wenig Bezahlung oder Anerkennung für Ihren Arbeitseinsatz?“[5]<br>„Leisten Sie häufig bzw. viele Überstunden ab?“[4]<br>„Ggf.: Haben Sie Schichtdienst und / oder häufig Nachtdienste?“[4]<br>„Machen Sie sich Sorgen um Ihren Arbeitsplatz oder Ihr berufliches Fortkommen?“	Ggf. berufliche Maßnahme, Stressmanagement-Training und / oder Psychotherapie[4]
**Familiärer Stress**[3]	„Haben Sie ernsthafte Probleme mit Ihrem / Ihrer Lebenspartner*in oder Ihrer Familie?“[3]	

Core-Net

Risikofaktoren	Mögliche Fragen	Empfehlungen
**Soziale Isolation** bzw. Mangel an sozialer Unterstützung[3]<br><br>(objektiv und subjektiv wahr-genommen)	„Leben Sie alleine?“[3]<br><br>„Vermissen Sie eine oder mehrere Personen, denen Sie vertrauen und auf deren Hilfe Sie zählen können?“[3]<br><br>„Haben Sie im vergangenen Jahr eine*n Angehörige*n oder Freund*in verloren?“[4]	Förderung des sozialen Netzwerkes[4]<br><br>z. B. durch Selbsthilfe-gruppen
**Psychische (Vor-) Erkrankung und Psycho-trope Medikamente[4]**	„Sind oder waren Sie schon einmal in einer psychotherapeutischen / psychiatrischen Behandlung?“<br><br>„Nehmen Sie stimmungsbeeinflussende Substanzen oder Medikamente ein?“<br><br>--<br><br>Psychotrope Substanzen inkl. Drogen, Johanneskraut, Baldrian etc.	Kontinuierliche fach-ärztliche Mitbehandlung sicherstellen[4]

CoRe-Net

Quellenverzeichnis:

1. Kroenke, K., Spitzer, R. L., Williams, J. B., Löwe, B. (2009). An ultra-brief screening scale for anxiety and depression: the PHQ–4. Psychosomatics, 50(6), 613-621.

2. Krupp, S., Seebens, A., Kasper, J., Willkomm, M., Balck, F. (2018). Validierung der deutschen Fassung des Six-Item Screeners. Zeitschrift für Gerontologie und Geriatrie, 51(3), 275-281.

3. Bundesärztekammer (BÄK), Kassenärztliche Bundesvereinigung (KBV), Arbeitsgemeinschaft der Wissenschaftlichen Medizinischen Fachgesellschaften (AWMF). Nationale VersorgungsLeitlinie Chronische KHK – Langfassung, 5. Auflage. Version 1. 2019 [cited: 2021-10-21]. DOI: 10.6101/AZQ/000419. www.khk.versorgungsleitlinien.de.

4. Albus, C., Waller, C., Fritzsche, K., Gunold, H., Haass, M., Hamann, B., Kindermann, I. (2018). Bedeutung von psychosozialen Faktoren in der Kardiologie–Update 2018. Der Kardiologe, 12(5), 312-331.

5. Visseren, F. L., Mach, F., Smulders, Y. M., Carballo, D., Koskinas, K. C., Bäck, M., Benetos, A. (2021). 2021 ESC Guidelines on cardiovascular disease prevention in clinical practice: Developed by the Task Force for cardiovascular disease prevention in clinical practice with representatives of the European Society of Cardiology and 12 medical societies with the special contribution of the European Association of Preventive Cardiology (EAPC). European Heart Journal, 42(34), 3227-33.

Alle Rechte vorbehalten:

© 2021 CoRe-Net: Kölner Kompetenznetzwerk aus Praxis und Forschung, Universität zu Köln I core-net@uk-koeln.de I core-net@uk-koeln.de

Alle Urheber- und Leistungsschutzrechte sind vorbehalten.

Diese Broschüre ist nur für den privaten Gebrauch bestimmt. Für alle Verwendungen, insbesondere Vorführung, Sendung, Bearbeitung und Vervielfältigung bedarf es einer speziellen Bewilligung soweit keine gesetzliche Lizenz besteht. Zuwiderhandlungen werden zivil- und straf-rechtlich verfolgt.

CoRe-Net
