# Supplementary material for: A tailored intervention for the detection of patients with coronary heart disease and mental or cognitive comorbidities in the German primary care setting: qualitative evaluation of implementation success
Source: BMC Health Serv Res. 2024 Nov 22;24:1454. doi: 10.1186/s12913-024-11841-z (PMC11585210; doi:10.1186/s12913-024-11841-z)
Supplement: Supplementary file 2 — Supplementary Material 2. [file 12913_2024_11841_MOESM2_ESM.pdf]

Screening für Demenz und Mild Cognitive Impairment

1. Subjektive Gedächtnisprobleme

„Haben Sie das Gefühl, dass Ihr Gedächtnis in letzter Zeit nachgelassen hat?"

➡ Bei positiver Antwort gefolgt von:

„Machen Sie sich deswegen Sorgen?"

2. Six-Item Screener[1]

„Ich nenne Ihnen 3 Dinge. Bitte warten Sie, bis ich alle 3 Wörter gesagt habe, und wiederholen Sie sie dann. Merken Sie sich die Wörter, denn ich frage Sie demnächst noch einmal danach. Bitte wiederholen Sie":

(1. Wort) – (2. Wort) – (3. Wort) z. B. „Auto - Blume - Ball"

Maximal 3 Lernversuche

➡ Alle 3 Wörter richtig wiederholt? ○ Ja ○ Nein

„Jetzt stelle ich Ihnen 3 Fragen":

„Welches Jahr haben wir?"	○ korrekt =1	○ nicht korrekt = 0
„Welchen Monat haben wir?"	○ korrekt =1	○ nicht korrekt = 0
„Welchen Wochentag haben wir?"	○ korrekt =1	○ nicht korrekt = 0

„Wie hießen die 3 Dinge, die Sie sich merken sollten?"

1. Wort	○ korrekt =1	○ nicht korrekt = 0
2. Wort	○ korrekt =1	○ nicht korrekt = 0
3. Wort	○ korrekt =1	○ nicht korrekt = 0

Anzahl der erreichten Punkte: ____ / 6

(Bei Erreichen von 5 oder 6 Punkten sind alltagsrelevante kognitive Störungen mit hoher Wahrscheinlichkeit ausgeschlossen).

Auffälligkeiten? ➡ Leitliniengerechte Versorgung und Ausgabe des Question Prompt Sheet

CoRe-Net

[1] Krupp, S., Seebens, A., Kasper, J., Willkomm, M., Balck, F. (2018). Validierung der deutschen Fassung des Six-Item Screeners. Zeitschrift für Gerontologie und Geriatrie, 51(3), 275-281.

Screening für psychische Störungen (Depression, Angst, Sonstige)

1. Offene Einstiegsfrage: „Wie fühlten Sie sich in den letzten Wochen in seelischer Hinsicht?"

2. Gesundheitsfragebogen für Patient*innen (PHQ-4)[1]:
„Wie oft fühlten Sie sich im Verlauf der letzten 2 Wochen durch die folgenden Beschwerden beeinträchtigt?"

		Überhaupt nicht	An einzelnen Tagen	An mehr als 50% der Tage	Beinahe jeden Tag
**Depression** (PHQ-2) = Summe der Items 1 und 2, Scores von ≥ 3 sind klinisch relevant.	1: Wenig Interesse oder Freude an Ihren Tätigkeiten	0	1	2	3
	2: Niedergeschlagenheit, Schwermut oder Hoffnungslosigkeit	0	1	2	3
**Angststörung** (GAD-2) = Summe aus den Items 3 und 4, Scores von ≥ 3 sind klinisch relevant.	3: Nervosität, Ängstlichkeit oder Anspannung	0	1	2	3
	4: Nicht in der Lage sein, Sorgen zu stoppen oder zu kontrollieren	0	1	2	3

3. Offene Anschlussfrage:
„Haben Sie noch weitere seelische Beschwerden außer den bisher genannten?"

Auffälligkeiten?
➡ **Leitliniengerechte Versorgung und Ausgabe des Question Prompt Sheet**

[1]Kroenke, K., Spitzer, R. L., Williams, J. B., Löwe, B. (2009). An ultra-brief screening scale for anxiety and depression: the PHQ–4. Psychosomatics, 50(6), 613-621.

CoRe-Net
