# Supplementary material for: A tailored intervention for the detection of patients with coronary heart disease and mental or cognitive comorbidities in the German primary care setting: qualitative evaluation of implementation success
Source: BMC Health Serv Res. 2024 Nov 22;24:1454. doi: 10.1186/s12913-024-11841-z (PMC11585210; doi:10.1186/s12913-024-11841-z)
Supplement: Supplementary file 3 — Supplementary Material 3. [file 12913_2024_11841_MOESM3_ESM.docx]

| **Key question (narrative prompt)** | **Memo for possible follow-up questions** |
| --- | --- |
| Initial question | |
| 1. **How did you come to take part in the study?** | - How did you hear about the project? - Why did it appeal to you? - What did you expect from your participation? |
| Intervention (application / feasibility) | |
| *[The introductory text describes the course of the intervention.]*  **In the past 6 months you have had the opportunity to apply the intervention. We would like to talk to you today about the experience you have gained during this time.**   1. **When you think about the use / application of the intervention. To what extent did you find this procedure feasible in daily practice?** | **General**   - Which components could be realised well/not well? - What problems or difficulties arose when using the intervention? - How well do you think the intervention can be integrated into existing work processes and structures? - To what extent has your use of the intervention changed over time (from the time of training until today)? |
|  | *If the intervention was not applied as specified:*   - How would you describe your typical approach? |
|  | *If not already described, ask about the application of the individual elements:*  **Did you apply the Trigger Question?**  If yes:   - At what point did you ask yourself the TQ? - With which patients did you ask the TQ? - How often do you repeat the TQ with the same patient? - What reminded you to ask the TQ?   If no:   - Why not? |
|  | **Have you used the screening tools?**  If yes:   - Which screening tool(s) did you use? - For which patients did you use the screening tools? - At what time did you use the screening tool?   If no:   - Why not? |
|  | **Have you used the information materials (booklet, coat pocket cards, tear-off sheets)?**  Wenn ja:   - Which one(s) did you use? - To read what? - How often did you use it?   If no:   - Why not? |
|  | **Did you give the QPS to patients?**  If yes:   - Which patients did you give the QPS to? - How did the handover of the QPS proceed? - How did the patients react to this?   If no:   - Why not? - Were the QPS displayed, e.g., in the waiting room? - How often did patients approach you directly with the QPS without you actively handing it out? (Take it from the waiting room)   If applicable: Would you have thought that this patient might be affected? |
|  | **How did the discussions with the patients about possible impairments go?**   - How did you experience the conversations with the patients? - What was the content of the conversation? - Were there any difficulties?   If applicable: Which ones? |
| Intervention (appropriateness, benefit, content) | |
| *[The introductory text states the aims of the study and the components of the intervention.]*   1. **Please tell us how you assess the benefits of the intervention?** | **General**   - To what extent did the intervention help to identify CHD patients with MCD?   What makes this possible?  What is still missing or what needs to happen to make this possible?   - Have you noticed a change in your own approach / way of thinking compared to before the study? - What is your assessment of this? To what extent does the approach initiate needs-orientated care for patients? |
|  | *If not already described, ask about the benefits of the individual elements:*  **TQ**   - How helpful did you find the TQ for this? - Why helpful? Or why not helpful? |
|  | **Screening tools**   - How helpful did you find the screening tools? - Why helpful? Or why not helpful? - Do you think there is a need for change? |
|  | **Information Materials (booklet, coat pocket cards, tear-off sheets)**   - How useful did you find the information materials you received from us? (booklet, coat pocket cards, tear-off sheets) - Why helpful? Or why not helpful? - Do you think there is a need for change? (booklet, coat pocket cards, tear-off sheets) |
|  | **QPS**   - What is your assessment of QPS for patients? (format, appearance, content) - In your opinion, is there anything that should be changed about the QPS? - To what extent has the QPS influenced physician-patient communication? - To what extent has the QPS contributed to joint decision-making in relation to the course of care? - How do you rate the benefits for patients? |
| Sustainability | |
| 1. **Will you continue the intervention after the end of the study?** | - Which components? - What are the reasons in favour? - What reasons are there against it? - What specifically would have to change for you to (continue to) use it? |
| Training course | |
| 1. **To what extent did the training course prepare you to use the intervention?** | - To what extent did the training course prepare you to use and implement the intervention? - What did you think of the training course concept? - What did you think of the content of the training course? - To what extent did the training course or the training course content have an influence on your practice? - What would have helped you to implement the intervention in your practice? |
| Final questions | |
| 1. How would you rate the intervention and supporting implementaion resources overall? Can you please summarise this in 2-3 sentences. | |
| 1. From your point of view, is there anything else that we have not yet discussed but that you would like to tell us? | |
| Summary and conclusion | |
| Thank you for your time and insights into your personal experiences.  *Stop recording* | |
