# Supplementary material for: A tailored intervention for the detection of patients with coronary heart disease and mental or cognitive comorbidities in the German primary care setting: qualitative evaluation of implementation success
Source: BMC Health Serv Res. 2024 Nov 22;24:1454. doi: 10.1186/s12913-024-11841-z (PMC11585210; doi:10.1186/s12913-024-11841-z)
Supplement: Supplementary file 4 — Supplementary Material 4. [file 12913_2024_11841_MOESM4_ESM.docx]

| **Exemplary extract from the cross-case matrix** | | |
| --- | --- | --- |
| **Outcome sustainability** | | |
| **Subcategory** | **Individual summary** | **Subcategory summary** |
| **Maintain individualised approach** | PCP1: Can imagine continuing the procedure after the end of the study. | The majority of PCPs indicated that they would maintain their individualised approach. |
|  | PCP 2: Describes wanting to continue using the intervention. |  |
|  | PCP 3: Indicates further use of the two questions on cognitive and psychological complaints for CHD patients and other patient groups. |  |
|  | PCP 4: Will continue to pay more attention to the comorbidity between CHD and MCD after the end of the study, documenting questions and signs and symptoms and conducting targeted tests - if there are indications. |  |
|  | PCP 7: Can imagine keeping the described procedure. |  |
|  | PCP 8: Will continue with the described procedure. |  |
|  | PCP 9: Have used the intervention beyond the intervention period and would like to continue using it. |  |
