# Supplementary material for: A tailored intervention for the detection of patients with coronary heart disease and mental or cognitive comorbidities in the German primary care setting: qualitative evaluation of implementation success
Source: BMC Health Serv Res. 2024 Nov 22;24:1454. doi: 10.1186/s12913-024-11841-z (PMC11585210; doi:10.1186/s12913-024-11841-z)
Supplement: Supplementary file 5 — Supplementary Material 5. [file 12913_2024_11841_MOESM5_ESM.docx]

| **Nummber of Quotation** | **Main- and Subcategory** | | **Approach** | **Quotation** |
| --- | --- | --- | --- | --- |
| **Appropriateness** | | | | |
| Q1 | Perceived Relevance of CHD and MCD Topics | | deductive | In itself, I find the topic of psychosomatics, the psyche in connection with physical illnesses, very important and very interesting, and we as primary care practitioners deal with it a lot.  PCP 8 |
|  | Changes related to MINI Intervention | | deductive |  |
| Q2 |  | Raise awareness of MCD and CHD among PCPs | inductive | So this topic was not so- maybe not so important for me earlier. I thought CHD is a difficult diagnosis. I didn't interpret it as an anxiety disorder or depression or or or. And, so I am actually more sensitised.  PCP 7 |
| Q3 |  | Reducing the stigmatisation of cognitive and mental impairment | inductive | Yes, for once, addressing it because many probably don't dare to and think they have to cope with it alone, and then there are of course appropriate support measures.  PCP 9 |
| Q4 |  | Spillover effects to other patient groups | inductive | With diabetics, it is also known that depression is a big topic. And I then also thought more about it in parallel. Now, independent of the study.  PCP 3 |
| Q5 |  | Discussions about possible cognitive and mental impairment | inductive | What I did more often as a result is simply to start a conversation. So, somehow asking out of the conversation, how are you dealing with it now, and what has changed for you now, or do you see life differently, or whatever.  PCP 6 |
| Q6 |  | Identification of MCD | deductive | So I could not identify cognitive burdens in CHD patients. With psychological burdens, where specific questions were asked, you could rather notice that patients reported fears or depressive phases.  PCP 5 |
| Q7 | Needs-orientated care | | deductive | Of course, I first tried to find a place for psychotherapy. That has NOT yet worked out, and I have already started antidepressants with one patient.  PCP 7 |
| Q8 | Perceived appropriate of MINI Intervention for the primary care setting | | deductive | We are the ones who accompany these patients over time. So it definitely belongs in the general practice setting, without any question. Yes, yes. But it would of course be helpful if the cardiologist also participated.  PCP 2 |
| Q9 | Intervention to all CHD patients was not considered appropriate | | inductiv | You have to see how you can apply it in a targeted way so that it is also effective. Not with a watering-can principle, but make it effective. PCP 10 |
| Q10 | Full screening of each patient with CHD | | inductiv | Because I already think it's good, at least with people with recent events, because I have already noticed a lot of uncertainty there, because it is simply a life-changing story.  PCP 9 |
| Q11 | Lack of availability of therapy places | | inductive | I think the bottleneck is that afterward there are always no therapists available or little support. Then you have identified it, and yes, and now?  PCP 9 |
| **Feasibility** | | | | |
|  | Barriers to use | | deduction |  |
| Q12 |  | Time constraints | deduktiv | In the routine of a general practice with a high patient volume, it is sometimes not practical. It is then difficult to implement. But otherwise, if you had the time for it, it would be easy. But under time pressure, it just fizzles out.  PCP 5 |
| Q13 |  | Acute health problems | inductive | It always depended a bit on the context of the consultation. Not every patient with CHD comes for consultation because of CHD, but sometimes for bronchitis or pneumonia. Even if you had asked the screening question and knew this is someone to watch out for, you didn't ask the other questions because it didn't fit the context of the practice. PCP 2 |
| Q14 |  | Building a relationship of trust | inductive | Many people need longer contact before they talk about any issues. And they don't trust us because they see a different doctor here every week. And that's also a point. With my regular patients, patients I know well, it works quite well, but with others, unfortunately not.  PCP 7 |
| Q15 |  | Remember the Intervention | inductive | It also decreased over time. That's also a problem. You're just in the daily rhythm. At the beginning, you're still so fully in the information loop. And then I paid more attention to it. And then you go on vacation for six weeks. Then, of course, everything is pushed aside a bit. And you're back in the older loop.  PCP 1 |
| Q16 |  | Lack of feedback | inductive | So you try to initiate it. How efficient that is, what impact it has on treatment, that is not always clear to me. You don't see the people that often either. Some you see once. And then they don't come back for months. And you don't follow up, I don't call again the one I sent to the psychologist.  PCP 2 |
| Q17 |  | Motivation | inductive | So I was definitely motivated at the beginning. But then I noticed that over the weeks it no longer became part of my standardized routine.  PCP 6 |
| Q18 |  | Patients under time pressure | inductive | But I have the impression that when a patient comes to me, he also feels under time pressure, just like I do. So he knows that he doesn't have much time here with me. And then he tries to deal with his problems or the prescription and everything he needs to do. But a patient has to relax so that you can talk about such issues at all.  PCP 7 |
| Q19 |  | Addressing patients for possible cognitive and mental impairment | inductive | At the beginning, it was a bit difficult to address it.  PCP 3 |
| Q20 |  | Patient reaction | inductive | I would say 90 per cent of the patients we addressed were always surprised. It was 'why should I'- or often the question was 'Why are you asking me this now?  PCP 4 |
|  | Potential for improvement | | deductive |  |
| Q21 |  | Shortening the screening | inductive | I think if you simplify it with very specific clear questions, which is not a lot of effort. I could imagine having a point whether there are psychological problems, yes or no, and differentiate which problems occur. As simple as possible.  PCP 5 |
| Q22 |  | Regular appointments |  | So maybe it will be easier if you actually include it as another question in the DMP questions. It would be no extra effort.  PCP 4 |
| **Acceptability** | | | | |
| Q23 | Structuring the workflow process | | inductive | It was really helpful to have it so well-structured in this training and then also to see it very structured at the beginning once or twice and then to discuss it with the patient sometimes a bit adapted from the wording in my head.  PCP 8 |
| Q24 | Did not fit | |  | So the questions are written in a way that sounds very good. And often it turns out a little bit different in conversation anyway. Not that I didn't like anything about it, but it just turns out more smoothly in conversation.  PCP 8 |
| Q25 | Information material | | deductive | Not leaving the patient alone but giving them something as a follow-up. So that they feel there is something structured. It's a very good idea.  PCP 10 |
| Q26 | Analogue format during consultation | | inductive | In conversation with the patient who is sitting across from you at close range, if I pull out a block and just start ticking boxes, they get the impression that this is now a standardized process. The personal attention and focus are distracted by these cards.  PCP 1 |
| Q27 | Physician Information System and pop-up windows | | inductive | So I think if it runs through the physician information system, the practice computer, and you see, that's the diagnosis, possibly a pop-up at the bottom right saying 'Psychological problem, sleep disorder?' then that would be a very good hint.  PCP 1 |
| Q28 | Training course | | deductive | It was a good meeting, which was very content-focused and really sensitized you to it, opened up a few facets that I hadn't realized before.  PCP 1 |
| Q29 | Reimbursement options | | inductive | It's a bit bitter, but it has to do with the cost issue, because if it is adequately compensated, then you also take the time for it. I can hardly open the space for how someone is doing emotionally and then be confronted with depressive symptoms and then say, okay, now the waiting room is full, now we have to move on immediately. And that, unfortunately, is connected.  PCP 8 |
| **Fidelity** | | | | |
| Q30 | MINI intervention used as intended | | deductive | I used more of my own wording to bring up the conversation, without always doing it standardized with some score.  PCP 6 |
|  | Using the TQ | | deductive | I have to honestly say, I didn't ask myself this trigger question.  PCP 9 |
|  | Use of screenings | | deductive | So I mainly used the cognitive screening instrument.  PCP 3 |
|  | Using information materials | | deductive | I found the material really great, and yet I almost didn't use it at all.  PCP 8 |
| **Sustainability** | | | | |
| Q31 | Maintain individualised approach in future | | inductive | I will incorporate it better into my everyday life, so the questions and document them in the medical record. And then do the tests specifically when I feel it's necessary.  PCP 4 |
| Q32 | Future use of information materials | | deductive | So we will continue to hand out the patient brochures.  PCP 5 |
